# The roles of microglia and astrocytes in neuroinflammation of Alzheimer’s disease

**DOI:** 10.3389/fnins.2025.1575453

**Published:** 2025-05-07

**Authors:** Jialin Han, Zhi Zhang, Pengfei Zhang, Qian Yu, Qian Cheng, Zhiming Lu, Shuai Zong

**Affiliations:** ^1^Department of Clinical Laboratory, Shandong Provincial Hospital, Cheeloo College of Medicine, Shandong University, Jinan, China; ^2^Department of Clinical Laboratory, Shandong Provincial Hospital Affiliated to Shandong First Medical University, Jinan, China

**Keywords:** Alzheimer’s disease, neuroinflammation, microglia, astrocyte, NLRP3 inflammasome

## Abstract

Alzheimer’s disease (AD) is a typical neurodegenerative disease, with the most highlighted pathologic changes identified in the *β*-amyloid peptide (Aβ) and neurofibrillary tangles (NFTs). Aβ cascade hypothesis, which has seemed to convincingly elucidate the AD pathogenic mechanism, is becoming increasingly disproved, indicating that it is no longer able to explain the emergence of AD entirely. Neuroinflammation offers an alternative explanation for the development of AD. This paper presents an overview of the influence of microglia and astrocytes on neuroinflammation of AD. We further examine the interplay between microglia and astrocyte and their roles as inflammatory mediators. It is hypothesized that targeting these molecular mechanisms associated with neuroinflammation and controlling risk factors may provide a viable therapeutic approach for AD.

## 1 Introduction

AD has become the leading cause of dementia, with a doubling of dementia prevalence in Europe and a tripling of worldwide prevalence by 2050 (Scheltens et al., 2021). Unless a new medical breakthrough, over 13.8 million Americans will be impacted by AD by 2060 (Alzheimers Dement, 2023). AD typically presents with a progressive decline in memory and cognitive function, eventually losing independence, which has shown an enormous impact on patient’s family and a higher economic cost to society. Caregivers’ stresses have also been increased (Bateman et al., 2012).

There are two major pathological alterations of AD, i.e., (1) extracellular lesions of AD refer to senile plaques, comprised by an accumulation of *β*-amyloid peptide (Aβ); (2) intracellular lesions are neurofibrillary tangles (NFTs), comprised by the excessively phosphorylated tau protein (p-tau) (Freitag et al., 2022). But it would be simplistic to define AD by these two biomarkers alone (Lecca et al., 2022). In recent years, a new AD characteristic has emerged that might provide a better understanding regarding the pathogenesis of AD. Multiple studies support that sustained neuroinflammatory also consist in AD brain when the existence of Aβ and NFT (Hong et al., 2016). Animal models and clinical research confirm the presence of neuroinflammation in the process of AD (Hou et al., 2021; Ohnishi et al., 2014; Wang et al., 2022). Additionally, studies consider neuroinflammatory may participate in synapse loss and dysfunction in AD brain before Aβ and NFT formation (Pan et al., 2022).

The main participants of neuroinflammation include microglia, astrocytes, oligodendroglia and the inflammatory factors released by these kinds of cells (Heneka et al., 2015). Meanwhile, microglia and astrocytes can further aggravating AD by interacting with each other in AD. Directly illustrate that glia and inflammatory factors are detrimental to AD is one-sided. Glial cells and inflammatory factors play different roles in different backgrounds (Liu et al., 2020a). Glial cells play a supporting role for brain under physiological conditions (Liu et al., 2023). Activated microglia, reactive astrocytes and dead oligodendrocytes aggravate AD (Sadick et al., 2022). Although the current study makes noteworthy progress, there remains controversy regarding whether the inflammatory response noted in AD is a result or cause of neurodegeneration. This review delves into the source and change of neuroinflammation, three kinds of glial cells and the inflammatory factors based on recent findings, demonstrating the potential of neuroinflammation as a viable therapeutic approach in AD treatment.

## 2 Glial cells in Alzheimer’s disease

### 2.1 Microglia

#### 2.1.1 Source of microglia

Microglia are the resident macrophages of the central nervous system (CNS) and are derived from primitive macrophages of the embryonic yolk sac. Erythro-myeloid progenitors (EMPs) develop into microglia, and the myeloid cell population in the CNS contains microglia as well as perivascular and meningeal-associated macrophages originated from the extra-embryonic yolk sac (Manouchehri et al., 2023). Macrophages that inhabit the meninges and perivascular spaces are referred to as border-associated macrophages (BAMs). It is generally difficult to distinguish myeloid cell subsets based on morphological characters, distribution areas, or the presence of certain cell-surface markers (Manouchehri et al., 2023). But myeloid cell subsets in the CNS perform different physiological functions. Microglia are mainly involved in synapse formation and synaptic transmission and plasticity (Parkhurst et al., 2013). Perivascular macrophages maintain brain glucose uptake by releasing vascular endothelial growth factors (VEGFs) (Jais et al., 2016). Leptomeningeal and perivascular macrophages regulate cerebral spinal fluid (CSF) flow dynamics (Drieu et al., 2022). When the CNS is damaged, monocytes that are derived from bone marrow can infiltrate the brain tissue and differentiate into highly phagocytic and inflammatory macrophages without contributing to the pool of resident microglia (Lazarov et al., 2023).

Neuronal apoptosis and the colony-stimulating factor 1 receptor (CSF1R) are both integral to the sowing of microglia precursors of the brain (Yang et al., 2021). Microglial development is highly dependent on CSF1R but not the colony-stimulating factor 1 (CSF1). As the ligand of CSF1R, interleukin 34 (IL-34) is also involved in the development of microglia (Greter et al., 2012). The level of transforming growth factor β1 (TGF-β1) is dependent on the upregulation of nuclear factor kappa-B (NF-κB) and its subunit cellular homolog of the v-Rel oncoprotein (c-Rel), which is a member of the nuclear factor kappa-B transcription factor family. Unfortunately, the programmed cell death protein 11 (PDCD11) deficiency leads to a decrease in c-Rel in the nucleus, ultimately causing the decreased level of TGF-β1 (Yang et al., 2021).

#### 2.1.2 Microglial physiology

Microglia with phagocytosis are innate immune cells in the CNS. They possess many immune functions, such as immune surveillance, immune defense, and phagocytosis (Prinz et al., 2019). In addition, microglia also play important functions related to the brain, depending on context, age, brain region, health, and metabolic needs (Bartels et al., 2020). Furthermore, microglia mediate synaptic pruning, regulate neuronal activity, provide trophic support, and produce effector molecules, including neurotropin-3, insulin like growth factor 1 (IGF1), and nerve growth factor (NGF), which are essential for the survival and development of neurons (Lazarov et al., 2023).

Microglial morphology is different during early postnatal and adult stages. In the prenatal and early postnatal stages, microglia display a generic macrophage-like and mobile amoeboid morphology, while in the adult stage, microglial morphology is altered to a highly ramified one, enabling microglia to survey their environment (Ormel et al., 2018; Parkhurst et al., 2013; Prinz et al., 2019) (see Table 1). These processes are often close to neuronal somata and dendritic spines (Parkhurst et al., 2013). Microglia possess diverse positive functions associated with the brain, including regulating the development of neural circuits in the immature brain and promoting the generation of neurons and learning in the adult brain (Eyo et al., 2018). The microglia–neuron junction is important for the management of injured neuron, with the process completed as follows: the neuronal cell body releases purine to combine with P2Y purinoceptor 12 receptor (P2Y12R) of the microglial membrane, and P2Y12R activation and microglial P2Y12 have a direct interaction with potassium channel voltage-gated potassium channel 2.1 (Kv2.1) clusters of neurons (Eyo et al., 2018; Miyamoto et al., 2013; Sharma et al., 2020).

**Table 1:** Comparison of microglia and astrocytes in AD.

Characteristics	Microglia	Astrocyte
Motility	Resting microglia move and survey the local microenvironment	Activated astrocytes are chemotactic to the damaged sites
Activation order	Early, microglia express TLRs 1 to 9	Late, astrocytes express TLR 4 in low levels
Morphology	Ameboid, activated microglia morphologically show enlarged cell bodies and shortened processes	Processes of reactive astrocytes become thicker and longer; the overall size of reactive astrocytes are similar to that of non-reactive astrocytes
Cellular phenotype	M1 (pro-inflammatory) phenotype; M2 (anti-inflammatory) phenotype	A1 (pro-inflammatory) phenotype; A2 (anti-inflammatory) phenotype
Main biomarker	Iba-1	Glial fibrillary acidic protein (GFAP)
Metabolization	Resting microglia depend mainly on OXPHOS; activated microglia favor glycolysis	Astrocyte is dominated by glycolysis; astrocytes possess low mitochondrial oxidative phosphorylation (OxPhos) activity; astrocytic OxPhos to degrade fatty acids (FAs) and maintain lipid homeostasis
Pathological function	Producing pro-inflammatory factors further activates inflammatory cells; inducing A1 astrocytes by secreting IL-1α, TNF, and C1q; aggravating Aβ and tau by activating NLRP3 inflammasome; destroying the synapses and neurons	Producing pro-inflammatory factors further activates inflammatory cells; activating microglia by secreting C3a; increasing the level of Aβ by expressing BACE-1, APP, and *γ*-secretase; leading to tau propagation by internalizing tau; destroying the synapses and neurons

#### 2.1.3 Microglia in Alzheimer’s disease

Reacting to the pathogenic infection, microglia are activated in the CNS (Zhang et al., 2018). Various types of stimuli activate different microglial phenotypes with varied functions (Cherry et al., 2014), including two main populations, the classically activated M1 (pro-inflammatory) phenotype and the alternatively activated M2 (anti-inflammatory) phenotype, with a spectrum of phenotypes between M1 and M2 (Zhang et al., 2018) (see Table 1). Several types of factors associated with pro-inflammation, such as tumor necrosis factor *α* (TNF-α), IL-1, reactive oxygen species (ROS) and reactive nitrogen species (RNS), are produced by M1 microglia (Cherry et al., 2014; David and Kroner, 2011). The generation of ROS and RNS is associated with microglial NADPH oxidase and inducible nitric oxidase (Cherry et al., 2014; Colonna and Butovsky, 2017). M2 microglia, with high levels of receptors related to phagocytosis, generate anti-inflammatory and trophic factors, such as IL-10, TGF-*β*, and brain-derived neurotrophic factor (BDNF) (Colonna and Butovsky, 2017). Therefore, microglia are a double-edged sword in AD.

By reprogramming microglial metabolism from oxidative phosphorylation (OxPhos) to glycolysis, Aβ acutely induces microglial activation (Baik et al., 2019) (see Table 1). Activation of Toll-like receptors (TLRs) in the microglia, especially Toll-like receptor 4, also induces the same metabolic change (Tannahill et al., 2013), which relies on the mammalian target of rapamycin-hypoxia-inducible factor-1a (mTOR-HIF-1a) pathway. This leads to a transformation of microglial phenotype to phagocytic, and the production and release of pro-inflammatory cytokines. Direct modulation of metabolic change influences the microglial phenotype related to both activation and deactivation of microglia (Baik et al., 2019). The existence of moderate neuroinflammation is positive to protect CNS, whereas prolonged inflammation aggravates the destruction of neurons (Crapser et al., 2020). Microglia decrease the Aβ aggregation by engulfing them and promote the formation of Aβ plaque by releasing inflammatory cytokines. The dual regulatory effects of microglia on amyloid-β (Aβ) pathology are primarily mediated through their dynamic phenotypic transition during mid-to-late AD stages. The early-stage protective phenotype characterized by Aβ phagocytosis via triggering receptor expressed on myeloidcells 2 (TREM2)-dependent clearance mechanisms (Keren-Shaul et al., 2017). Activated microglia progressively shifts toward a chronic pro-inflammatory state in advanced AD stages (Ising et al., 2019). Activated NOD-like receptor thermal protein domain associated protein 3 (NLRP3) inflammasome of microglia inhibits the elimination of Aβ, thus aggravating the Aβ plaque and elevating the progress of AD (Li et al., 2023).

**Figure 1:**
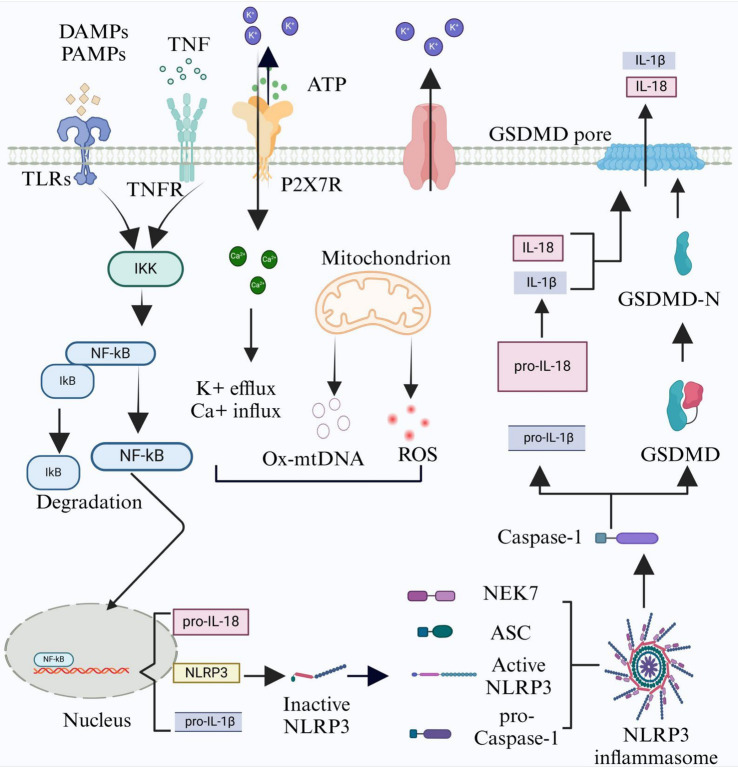
The mechanism of canonical NLRP3 inflammasome activation. The activation of canonical NLRP3 inflammasome is completed in two steps, the priming and the activation of NLRP3 inflammasome. During the priming step, ligands (i.e., DAMPs, PAMPs, and TNF) link to matching receptors, i.e., toll-like receptors (TLRs) and TNFR, to induce the expression of pro-IL-1β, pro-IL-18, and NLRP3 via NF-κB pathway. During the activation step, a variety of molecular and cellular events, such as K^+^ efflux, Ca^+^ influx, mitochondrial dysfunction, release of reactive oxygen species (ROS) and oxidative mitochondrial DNA ox-mtDNA, conversion of inactive NLRP3 to active NLRP3 and furthering NLRP3 inflammasome assembly. Activated NLRP3 inflammasome induces pro-caspase-1 self-cleavage to generate caspase-1, which is proteolytically active. Activated caspase-1 promotes the formation of the proinflammatory cytokines interleukin 1β (IL-1β) and interleukin 18 (IL-18). In addition, activated caspase-1 also cleaves and separates gasdermin D (GSDMD) and releases its N-terminal, which forms plasma membrane pores, triggers the release of IL-1β and IL-18, and induces pyroptosis. This figure is created by biorender.

The activation of inflammasome is completed in two steps. First, the priming of NLRP3 inflammasome is essential for its activation. Multiple transcriptionally active signaling receptors are involved in the priming of microglia. TLRs and tumor necrosis factor receptor (TNFR) combine with their ligands, leading to the activation of NF-κB (Bauernfeind et al., 2009; Franchi et al., 2009). Priming plays two roles, including up-regulating the expression of the inflammasome components (i.e., NLRP3, caspase 1, and pro-IL-1β) and inducing the post-translational modifications (PTM) of NLRP3, which keeps NLRP3 in inactive but normal signal state. Second, after identifying NLRP3 activators, NLRP3 inflammasome is fully activated and assembled (Swanson et al., 2019). When microglia largely engulf Aβ, microglial lysosome exhibits swollen morphology during the internalization and undergoes structural damage. Damaged lysosome releases lysosomal protease cathepsin B to activate NALP3 inflammasome (Halle et al., 2008). ATP in damaged cells activates P2X purinoceptor 7 receptor (P2X7R), a ligand binding non-specific ion channel, and then NLRs detect cytosolic ion fluxes. NLRs, such as NALP3, promote the activation of caspase-1, which cleaves and activates IL-1 family cytokines (Ransohoff and Brown, 2012). The activation of caspase-1 refers to the self-cleavage of pro-caspase-1, generating caspase-1 that is proteolytically active (Huang et al., 2021). Activated caspase-1 cleaves pro-IL-1β and pro-IL-18 into their bioactive forms, i.e., IL-1β and IL-18, respectively (Guo et al., 2015). NF-κB is an essential positive transcription regulator of gasdermin D (GSDMD) (Liu et al., 2017). In the typical environment, the C-terminal of GSDMD restrains the activity of the N-terminal. However, N-and C-terminals of GSDMD can be cleaved and separated by active caspase-1. Then, the N-terminal of GSDMD, forming plasma membrane pores, triggers the liberation of IL-1β, IL-18, and apoptosis-associated speck-like protein containing a C-terminal caspase recruitment domain (ASC), and induces pyroptosis (Lei et al., 2018) (see Figure 1).

Both Aβ and tau induce the activation of NLRP3 inflammasome, which in turn aggravates Aβ and tau. For Aβ, ASC combines with Aβ to increase the formation of Aβ oligomers and fibrils. For tau, active NLRP3 inflammasome increases the glycogen synthase kinase-3β (GSK-3β) kinase activity and aggravate tau hyperphosphorylation (Ising et al., 2019; Venegas et al., 2017).

### 2.2 Astrocyte

#### 2.2.1 Astrocytic physiology

Most neurons and glia can be produced by radial glia that are CNS progenitors, either directly or through intermediate progenitors. In the completion of gliogenesis, grown astrocytes can be differentiated directly from radial glia, which are concurrently produced during the final stages of neurogenesis (Allen and Lyons, 2018).

The most abundant glial cells of CNS are astrocytes, which are ubiquitous in brain and are separable among several CNS regions (Endo et al., 2022). Astrocytes and others, such as microglia and oligodendrocyte, have different morphological structures, with astrocytes exhibiting highly complicated, dense, and sponge-like morphology (Nagai et al., 2021) (see Table 1). Furthermore, the morphological and functional variations of astrocytes in different CNS regions are determined by genes and local tissue environment (Endo et al., 2022). According to the volume of glial filaments and the branching of processes, astrocytes grouped into two categories, protoplasmic astrocytes of gray matter (GM) and fibrous astrocytes of white matter (WM), which have essential differences in morphology. In particular, protoplasmic astrocytes have high branching, characterized by round shaped somata and a small quantity of glial fibrillary acidic protein (GFAP), while fibrous astrocytes have poor branching, characterized by longer somata with slim and long processes (Kohler et al., 2023).

Astrocytes that are generally stelliform cells with thousands of processes interacting with synapses and other cells in the brain and exhibiting a range of functions (Allen and Lyons, 2018). Astrocytes regulate the functions of synapses and neurons, as well as ion, neurotransmitter homeostasis, synaptic modulation, and metabolism. Protoplasmic astrocytes are interconnected with the presynaptic and postsynaptic compositions of the synapse, which is defined as the tripartite synapse. Astrocytes also contact with vasculature to provide support to neuronal metabolism (Halassa et al., 2007). A sign of brain function is the tight spatiotemporal coupling between synaptic activity and local blood flow. Astrocytes have shown a strong capacity of local vascular regulation. At least in part, the active synapses release glutamic acid to combine with metabotropic glutamate receptor 5 (mGluR5) receptor in the astrocytes, ultimately causing the increased level of astrocytic Ca^2+^, which is related with vasodilation in astrocytic endfeet (Takano et al., 2006). Brain relies on glucose as its main source of fuel, and astrocytes provide glucose to neurons via their contact with blood–brain barrier (BBB) (Cunnane et al., 2020). Thus, astrocytes provide metabolic support in response to the level required for neuronal activity.

This synaptic relation also confers other functions, including clearing undesired synaptic transmitters. The presynaptic nerve terminal releases synaptic transmitters to act on postsynaptic receptors. Plasma-membrane transporters for glutamate show a high density in the astrocyte membrane, driving the transmitters entry into the astrocytes through Na^+^ and K^+^ gradients. This action of astrocytes quickly clears synaptic transmitters from synapse to maintain the fidelity of synaptic transmission (Armbruster et al., 2022). Furthermore, astrocytes clear glutamate by converting glutamate to glutamine catalyzed by glutamine synthetase (GS) and releasing glutamine into the extracellular space by specific transporters. Neurons express transporters that uptake glutamine and convert glutamine back to glutamate by phosphate-activated glutaminase, which catalyzes the presynaptic membrane to release glutamate that acts on the postsynaptic receptors. This process is called the glutamate-glutamine cycle, enabling the continuous synaptic transmission (Tani et al., 2014) and relieving neurons of an energetic burden (Cunnane et al., 2020).

Astrocytes respond to neuronal signals by releasing gliotransmitters. ATP is almost entirely classified as a paracrine messenger, because it is a ubiquitous glial transmitter. ATP can connect with postsynaptic P2X receptors (of the purinergic receptor family), which elevate the level of postsynaptic Ca^2+^. Then, the calcium-dependent phosphatidylinositol 3-kinase (PI3K) activation drives the insertion of *α*-amino-3-hydroxy-5-methyl-4-isoxazole-propionic acid receptors (AMPARs) to regulate the synaptic transmission (Gordon et al., 2005).

#### 2.2.2 Astrocyte in Alzheimer’s disease

With the development of AD, astrocytes are transformed from homeostatic state towards reactive state, which defines the reactive astrocytes. These cells express a higher level of GFAP compared with the normal astrocytes. The most common marker of reactive astrocytes is GFAP (Escartin et al., 2021), which is morphologically different from the normal astrocytes. The GFAP-positive processes of reactive astrocytes become thicker and longer, while the overall size of reactive astrocytes is similar to that of non-reactive astrocytes (Wilhelmsson et al., 2006). Fatty acids (FAs) are essential components of almost all types of lipids in the brain. But excess FAs can lead to lipotoxicity. Therefore, FAs need to be finely regulated. Although astrocytes have shown low mitochondrial OxPhos activity, the degradation of FAs requires the astrocytic OxPhos (see Table 1). Overload of FAs leads to mitochondrial export of lots of acetyl-coenzyme A, boosting the activation of signal transducer and activator of transcription (STAT3) and leading to the astrocytic reactiveness and the release of pro-inflammatory cytokines (Mi et al., 2023).

Neurotoxic factors associated with AD, such as Aβ and cytokines, robustly up-regulate the number of calcineurin (CN) of astrocytes. CN promotes the activated phenotype of astrocytes by dephosphorylating the nuclear factor of activated T-cells (NFAT) transcription factors (Furman et al., 2012). Astrocyte activation in the CNS is heterogeneous (Zamanian et al., 2012). Reactive astrocytes can be divided into two opposing types, which can be induced by different stimuli, e.g., ischaemia activates A2 astrocytes and neuroinflammation induces A1 astrocytes (Liddelow et al., 2017). These subtypes diverge in ultrastructural morphology, molecular marker expression, and disease-modifying functions within AD pathogenesis.

Reactive A1 astrocytes are characterized by soma swelling, process retraction, and significantly diminished process complexity. Correspondingly, soma hypertrophy coincides with enhanced release of pro-inflammatory factors, reflecting the pro-inflammatory nature of A1 astrocytes (Althammer et al., 2020) (see Table 1). And single-cell transcriptomic profiling reveals that A1 astrocytes exhibit transcriptional divergence from A2 subtypes, such as the complement proteins 3 (C3). The C3 gene is highly upregulated in A1 astrocytes but absent in A2 astrocytes. This binary expression pattern positions C3 as a specific molecular signature for A1 subtype (Liddelow et al., 2017). Functionally, A1 astrocytes drive neuroinflammation through NF-κB-dependent mechanisms (Sun et al., 2024). A*β* oligomers activate the canonical NF-κB pathway in astrocytes via Toll-like receptor 4, triggering nuclear translocation of p65/p50 heterodimers. This transcriptional reprogramming promotes the expression of target genes encoding pro-inflammatory factors (IL-1, IL-6, TNF-*α*, and cyclooxygenase 2 (COX-2)) and C3 (Perez-Nievas and Serrano-Pozo, 2018). These inflammatory mediators are involved in neurological damage. For example, C3 can combine with C3a receptors on neuronal membrane, destroying neuronal dendritic structure and synaptic functions (Perez-Nievas and Serrano-Pozo, 2018).

Comparative ultrastructural analyses reveal that A2 astrocytes exhibit distinct morphological features including polarized soma orientation, elongated processes, and intricate terminal branches compared to A1 astrocytes (Althammer et al., 2020). Transcriptomic profiling identifies S100 calcium-binding protein A10 (S100A10) as a cardinal molecular marker of the A2 astrocytes, with functional studies demonstrating its neuroprotective role through trophic support mechanisms (Guo et al., 2020; Liu et al., 2020b). The A2 phenotype demonstrates functional role in inflammation resolution through IL-10 and TGF-*β*, which establish an immunoregulatory microenvironment conducive to tissue repair (Sofroniew, 2015). Notably, these cells synthesize elevated levels of neurotrophic factors, including cardiotrophin-like cytokine factor 1 (CLCF1) and leukemia inhibitory factor (LIF), which orchestrate synaptic remodeling through activation of the JAK–STAT signaling cascade (Zamanian et al., 2012). Furthermore, the ubiquitin modifying protein A20, synthesized by A2 astrocytes, functions as a indirect anti-inflammatory mediator. TNF-α signaling induces the activation of NF-κB in reactive astrocytes and A20, which suppress the activation of NF-κB. Therefore, A20 produced by astrocytes is a critical anti-inflammatory regulator (Wang et al., 2013). To summarize, both the beneficial functions of astrogliosis and its harmful effects are regulated by specific signaling mechanisms.

The membrane of astrocytes contains the low-density lipoprotein receptor (LDLR), which promotes the absorption of lipoprotein particles by binding apoE and apoB-100. In the AD, reactive astrocytes are localized around Aβ plaques. The LDLR of astrocytes combine with Aβ to modulate the Aβ uptake and clearance (Basak et al., 2012). However, the initial Aβ can induce astrocytes to generate Aβ. In the terminal stage of AD, the activated astrocytes around Aβ plaques express amyloid precursor protein (APP), *γ*-secretase, and β-site amyloid precursor protein cleaving enzyme 1 (BACE-1), to increase the level of Aβ (Grolla et al., 2013; Hartlage-Rubsamen et al., 2003). In addition, astrocytes facilitate Aβ clearance through lipoprotein receptor-related protein 1 (LRP1)-mediated pathways, but significant downregulation of LRP1 expression results in impaired Aβ removal efficiency in AD (Ma et al., 2018). Reactive astrocytes move to around both Aβ plaques and NFTs. The level of astrocytosis is not only increased with Aβ plaques, but also changed with the burden of NFTs (Serrano-Pozo et al., 2011). Reactive astrocytes are essential to trigger Aβ-induced tau phosphorylation, while astrocytes that are GFAP-positive can internalize tau, resulting in tau propagation (Bellaver et al., 2023).

## 3 Microglia-astrocyte crosstalk

### 3.1 Protective microglia–astrocyte interaction

Although the origins of both microglia and astrocytes are different, with microglia originated from yolk sac at embryonic phase (Ginhoux et al., 2010) and astrocytes originated from ectodermal neuroepithelium (Verkhratsky and Nedergaard, 2018), the anatomical positions of both microglia and astrocytes are quite close.

Different glial cells have varied functions. Astrocytes participate in the structure of the vascular unit of the CNS (Jackson et al., 2022), with a strong capacity of local vascular regulation (Takano et al., 2006). Resting microglia have ramified motile processes and explore the surrounding environment by extending and withdrawing the processes in the adult brain. Therefore, when there are pathological changes in homeostasis, microglia are considered the first responder to these variations (Bernier et al., 2020). Microglia and astrocytes can coordinate with each other in some of the functions of the brain (Clark et al., 2021).

The microglia-astrocyte interactions can be reflected in synaptic pruning. Synapses expressing C1q and C3 induce microglia to move to the vicinity of the synapses, with microglial C3R bound to C1q and C3, pushing microglia to engulf synapses and complete cleavage of synapses (Lehrman et al., 2018). Astrocytes clear unnecessary excitatory synaptic connections by multiple epidermal growth factor-like domains 10 (MEGF10), which is essential for keeping circuit connection and thereby protecting cognitive function (Lee et al., 2021).

Both microglia and astrocytes protect synapse formation and connection. In order to protect synapse itself, normal synapses expressing CD47 bind to microglial CD47 receptor signal regulatory protein alpha (SIRPα), preventing aberrant phagocytosis (Lehrman et al., 2018). Astrocytes modulate the timing and degree of synapse formation by secreting various molecules. For example, astrocytes guarantee the correct construction of thalamocortical synapses by secreting hevin (Risher et al., 2014). Astrocytes can secrete IL-33 to combine with IL1R of microglia, promoting synaptic engulfment (Vainchtein et al., 2018).

In addition, microglia and astrocytes also interact in cleaning dying neurons. Astrocytes move through their distal processes, rather than the soma, to engulf the dendritic apoptotic bodies of dead neurons, whereas microglia engulf the soma and apical dendrites of dying neurons by migrating to death neurons. The synergy between microglia and astrocytes is tightly controlled by two receptor tyrosine kinases, Axl receptor tyrosine kinase (Axl) and Mer tyrosine kinase (Mertk) (Damisah et al., 2020).

Astrocytes can complement the phagocytic function of microglia. In the physiological situation, quiescent microglia survey the surrounding microenvironment and activate the microglia engulf debris (Rigato et al., 2011). Although the resting astrocytes express phagocytosis-related receptors, such as Axl and Merkt (Miner et al., 2015; Zhou et al., 2021), they are in the standby state. After microglia are damaged, astrocytes are activated to clean damaged microglial and neuronal debris. Meanwhile, the activated astrocytes release pro-inflammatory factors, causing a parenchymal cytokine storm and leading to neuroinflammation (Konishi et al., 2020). Furthermore, crosstalk between microglia and astrocytes can promote the proliferation of microglia (Kim and Son, 2021).

### 3.2 Microglia-astrocyte crosstalk in Alzheimer’s disease

Microglia and astrocytes bidirectionally communicate during neurodegeneration. The microglia-astroglia crosstalk can be achieved by extracellular vesicles (EVs), which are produced by cells containing biological molecules, such as small RNAs and proteins. EVs can be transferred between cells (Neckles et al., 2019). Microglia can produce EVs that contain a set of proteins activate astrocytes, while microglia stimulated by ATP produce EVs, which show high impact on the activation state of astrocytes (Drago et al., 2017). EVs that are produced by astrocytes in turn influence the microglial activation. MicroRNAs play dual roles in the activation of microglia. For example, astrocytes release miR-873a-5p in the exosomes to decrease the microglia-mediated neuroinflammation by restraining the activation of extracellular signal-regulated kinase (ERK) and NF-κB (Long et al., 2020), while miR-138 in the EVs of astrocytes can activate microglia (Liao et al., 2020). Moreover, EVs produced by astrocytes are taken up by microglia, which up-regulate the expression of lincRNA-Cox2 to destroy the phagocytic function of microglia (Hu et al., 2018).

In the AD, microglia are activated earlier in comparison with astrocytes. The pattern recognition receptors (PRRs), such as TLRs, participate in the activation of cells. Microglia express TLRs 1 to 9, whereas astrocytes express TLR3 in high level but TLRs 1, 4, 5, and 9 in low levels (Jack et al., 2005). Lipopolysaccharide (LPS) is the TLR4 ligand. Activated TLR4 induced by LPS triggers the production of pro-inflammatory factors, such as TNF-*α*, IL-1*β*, prostaglandin E2 (PGE2), and nitric oxide (NO). These cytokines cause neurodegeneration and cognitive deficits (Kwon and Lee, 2022; Zhao et al., 2019). Microglia with high expression of TLR4 can be activated by LPS, while astrocytes with low expression of TLR4 but high expression of TLR3 can be activated under LPS stimulation. This is because that A1 astrocytes can be induced by IL-1α, TNF, and C1q secreted by LPS-activated microglia, indicating that activated microglia are required for the activation of astrocytes (Liddelow et al., 2017). Therefore, microglia and astrocytes activate each other in AD, increasing the generation of pro-inflammatory factors, damaging the phagocytosis of both microglia and astrocytes, aggravating neuroinflammation, ultimately leading to disorders of cognition and memory loss.

## 4 Inflammatory factors in Alzheimer’s disease

### 4.1 Pro-inflammatory factors

The production of various mediators of inflammation through different cellular pathways requires inflammatory transcription factors. In particular, the canonical Janus kinase (JAK), a type of protein-tyrosine kinase, is activated through the binding of interferon-*γ* (INF-γ) to its receptor IFNγR. Subsequently, JAK phosphorylates tyrosine of STAT1 to activate STAT1, promoting the translocation of STAT1 to the nucleus. STAT1 combines with conserved IFN*γ* activation site (GAS) DNA elements in the nucleus to activate interferon-stimulated genes (ISGs), which encode MCH-II and chemokines. This process promotes the production of pro-inflammatory cytokines (i.e., IL-1*β*, TNF-α, and IL-6) and NO (Ivashkiv, 2018; Zhang et al., 2020). In addition, NF-κB transcription factor is important for the production of pro-inflammatory factors in microglia and astrocytes of AD. Generally, inhibitor of kappaB (IκBs) as an NF-κB inhibitor link to NF-κB dimers, which sequester NF-κB in the cytoplasm (Sun and Ley, 2008). Thus, IκBβ inhibits the activation of NF-κB (Rao et al., 2010). Various immune receptors, such as the TLRs, IL-1R, and TNFR, activate the IκB kinase (IKK) complex to phosphorylate IκBs and trigger the degradation of IKBs. Then, the activated NF-κB is translocated to nucleus to promote the expression of genes encoding inflammatory factors, such as IL-1, IL-6, TNF-α, and ROS (Sun and Ley, 2008). Both microglia and astrocytes contain TLRs, indicating that NF-κB is important for neuroinflammation of microglia and astrocytes (Jack et al., 2005; Kawai and Akira, 2007).

Different pro-inflammatory factors play varied roles. IL-1 directly up-regulates the expression of mitogen-activated protein kinase p38 (MAPK-p38) mRNA, while activated MAPK-p38 promotes the phosphorylation of tau and neurofibrillary production (Sheng et al., 2001). IL-1 also stimulates the production of other pro-inflammatory cytokines, such as TNF-α and IL-6 (Dionisio-Santos et al., 2020). TNF-α up-regulates the expression of *β*-secretase, which is necessary for Aβ generation, suggesting the effect of TNF-α on AβPP processing and Aβ generation (Paouri et al., 2017). Increased levels of NO can stimulate nitration of many proteins, such as nitration at tyrosine 10 [3NTyr(10)-Aβ]. Nitration of Aβ accelerates its own accumulation (Kummer et al., 2011). Both superoxide anion and NO promote the generation of more cytotoxic agents, leading to neuronal cell death (Shabab et al., 2017). IL-6 is involved in the activation of MAPK signaling pathway, which promotes the increase in Egr-1 expression and elevates the p35 levels. IL-6 also induces tau hyperphosphorylation by activating cdk5/p35 complex (Quintanilla et al., 2004). In addition, inflammatory cells can produce chemokines, COX-1, and COX-2, which promote the development of AD.

### 4.2 Anti-inflammatory factors

Neuroinflammation is a significant mechanism in the occurrence and development of AD. Pro-inflammatory factors can aggravate AD, whereas both microglia and astrocytes can produce anti-inflammatory factors. During brain injury, local neurons promote the transformation of microglia to M2 by releasing IL-4. In order to relieve neuroinflammation, M2 microglia can increase the augmentation of phagocytosis and proteolysis of dead cells and pathological proteins (Zhao et al., 2015). M2 microglia also produce anti-inflammatory factors, such as IL-10 and TGF-*β*. Injured neurons can release lipocalin-2 (LCN2), which guides the transformation of microglia and astrocytes into pro-recovery phenotypes. LCN2-activated microglia release IL-10, while LCN2-activated astrocytes release BDNF and thrombospondin-1 (TSP-1) (Xing et al., 2014).

Different anti-inflammatory factors have not only shared but also different functions. IL-4 can promote the expression of anti-inflammatory factors, such as IL-10, TGF-β, and arginase-1, and reduce neuroinflammation (Dionisio-Santos et al., 2020). IFN-*γ* can stimulate astrocytes to secrete thiols and lactate. Both IFN-γ and IL-4 mediate cysteine release from astrocytes in a dose-dependent manner (Garg et al., 2009). IL-10 not only inhibits the release of pro-inflammatory cytokines, such as IL-1 and IL-6, but also suppresses caspase-3-mediated neuronal apoptosis. Although IL-10 is not involved in the reduction of beta-amyloidosis in the brain, it enhances the vascular transport of Aβ (Kiyota et al., 2012). As an anti-inflammatory factor, TGF-β1 reduces the amyloid pathology and neuronal loss. However, the level of TGF-β1 is decreased in AD (Shen et al., 2014; Tesseur et al., 2006).

There is an anti-inflammatory pathway in glial cells of brain. Under the homeostatic conditions, Nuclear factor erythroid 2-related factor 2 (Nrf2) is connected with the bric-à-brac, tramtrack, and broad-complex (BTB) domain-containing protein Keap1. Thus, Keap1 keeps the inactive Nrf2 in the cytoplasm and tightly regulates the proteolysis of Nrf2 (Cullinan et al., 2004). When several highly reactive cysteines in Keap1 are modified by electrophilic molecules, the structure of Keap1 changes. Structurally altered Keap1 is first separated from Nrf2 and then is degraded. Nrf2 is activated and translocated to nucleus (Cuadrado et al., 2019). Keap1 restrains phosphorylation of IKKβ to block the activation of IKK β. Thus, Keap1 can prevent NF-κB translocation to nucleus, ultimately inhibiting neuroinflammation and promoting anti-inflammation (Kim et al., 2010). Furthermore, Keap1 is involved in other anti-inflammatory pathways, e.g., active Nrf2 binds p300 by competing with NF-κB to inhibit the expression of genes associated with pro-inflammatory factors (Kim et al., 2013). Nrf2 activation not only suppresses neuroinflammation but also mitigates cognitive deficits by reducing intracellular reactive oxygen species (ROS) accumulation (She et al., 2024).

## 5 Prevention and treatment

### 5.1 Prevention of Alzheimer’s disease

Many risk factors are involved in the initial developmental stages of AD. Pathological changes, such as Aβ, NTFs, and neuroinflammation, are important causes of AD. In addition, various controllable factors, such as diabetes, obesity, sleep disorder, and lack of exercise, also influence AD. Here, the adjustment of diet, physical activity, and sleep, which is considered effective strategy to prevent AD, is primarily introduced.

Western dietary pattern is associated with cognitive decline, due to its generally high levels in not only calories, sugars, but also trans and saturated fats. Therefore, the intake of fats and carbohydrates increases the severity of AD (Alles et al., 2019). High-fat diet (HFD) raises the level of β-site amyloid precursor protein cleaving enzyme 1 (BACE-1), the concentration of soluble Aβ, and the glial reactivity, indicating that HFD can aggravate memory deficits (Busquets et al., 2017). High-sugar diet causes cognitive decline and brain function injury. HFD and high-sugar diet can decrease the expression of BDNF, a type of neurotrophin associated with synaptic plasticity as well as the survival and function of neurons, which is correlated with memory deficits (Chong et al., 2019). Due to the aggravated development of AD by both HFD and high-sugar diet, it is important to take into consideration of a balanced diet. However, the requirements for a balanced diet in different countries or regions could be varied based on the environmental and genetic alterations. The specific nutritional combinations need further exploration.

Exercise slows the progression of cognitive injury and ameliorates cognitive function and memory of humans (Ogino et al., 2019). Studies have revealed the association between exercise and AD. For example, in aged mice, wheel running exercise enhances survival of new neurons and microglial phenotype transformation to neuroprotection. Furthermore, physical exercise improves the cognitive function (Jiang et al., 2017; Kohman et al., 2012), e.g., voluntary aerobic activity can augment the BDNF expression of the hippocampus, ultimately improving memory and learning functions (Wang and Holsinger, 2018).

The pathogenic mechanism underlying the disturbances of the circadian system and AD share some general features. Dyssomnia is a significant player on the onset and progression of AD. Sleep deprivation (SD) accelerates the structural change of tau, leading to significant decrease of the tau solubility. The development of tau pathology results in synaptic deficits, leading to biochemical and functional destruction of synapses (Di Meco et al., 2014). Restorative function of sleep increases the interstitial space, enhancing the exchange speed between CSF and cellular interstitial fluid. In turn, exchange of CSF with interstitial fluid improves the clearance of Aβ, ultimately elevating the memory function (Xie et al., 2013).

### 5.2 Anti-inflammatory treatment of Alzheimer’s disease

Neuroinflammation accelerates the development of AD. Both inflammatory cells (e.g., microglia and astrocytes) and inflammatory factors (e.g., IL-1 and TNF-*α*) released by inflammatory cells aggravate the pathology of AD and disruption of synapses, causing memory deficit. Therefore, anti-inflammatory treatment is a commonly used therapeutic strategy of AD. Anti-inflammatory treatments include non-drug therapy and drug therapy.

For the non-pharmacological treatment, the change of lifestyle can alleviate the neuroinflammation. Low-fat diet (LFD) partially reverses the neuroinflammatory response, Aβ accumulation, tau phosphorylation, and cognitive impairment caused by HFD (Walker et al., 2017). Exercise increases the expression of BDNF, inhibiting neuroinflammation and decreasing the release of IL-1β and IL-10 (Dallagnol et al., 2017). Non-steroid anti-inflammatory drugs (NSAIDs) alleviate symptoms of some patients of AD by inhibiting COX and other mechanisms (O'Bryant et al., 2018). As the inhibitors of COX, NSAIDs inhibit the conversion of arachidonic acid to pro-inflammatory PGs. There are at least two COX isoforms, COX-1 and COX-2. Accordingly, NSAIDs are divided into two groups, i.e., non-selective COX-inhibitors (i.e., aspirin and ibuprofen) and COX-2 selective inhibitors (i.e., meloxicam and etodolac) (Imbimbo et al., 2010). Different NSAIDs may use different mechanisms to alleviate symptoms, e.g., indomethacin inhibits the activation of NF-κB to reduce the amyloid pathology. In contrast, nimesulide is not effective for both Aβ and NF-κB (Sung et al., 2004).

One of the pharmacotherapeutic treatments of inflammation in AD is to directly target inflammatory factors. Anti-inflammation can be achieved by the use of monoclonal antibodies and synthetic inhibitors against inflammatory factors. TNF-α is a kind of important pro-inflammatory factor associated with AD. Infliximab, a monoclonal antibody with high affinity for human TNF, is used to bind with TNF to reduce the TNF level, tau phosphorylation, and Aβ plaques, leading to improved visual recognition memory (Orti-Casan et al., 2019). TNF-α inhibitor (etanercept) significantly reduces the level of p-tau and microgliosis and improves neuronal health (Ou et al., 2021). Between the two types of TNFR, TNFR1 is involved in neuroinflammation of AD, while TNFR2 is involved in neuroprotection. Therefore, TNFR1 antagonist and TNFR2 agonist can improve memory and cell viability and decrease neuroinflammation (Orti-Casan et al., 2019).

Inflammation in AD can also be treated with antioxidants. In AD, the elevated level of ROS in microglia causes activation of inflammation, and antioxidants can inhibit neuroinflammation (Zhao et al., 2021). Both neuroinflammation and oxidative stress are the main pathogenic mechanisms in AD, with both interacting with each other to aggravate their severity and promote the development of AD.

### 5.3 Therapy associated with microglia

Microglia are the primary immune cells of the CNS and play a key role in the pathogenesis of AD. Pathogen-associated molecular patterns (PAMPs) and damage-associated molecular patterns (DAMPs) can activate microglia. Activated microglia have shown different phenotypes with varied functions. In different stages of AD, microglial phenotypes and functions change. Overactivated microglia aggravate Aβ, NFTs, and neuroinflammation. Targeted microglial therapy is one way to improve disease. Interventions on microglia are conducted from two aspects, i.e., inhibiting microglial activation and pro-inflammatory response, and modulating microglial phenotype.

The inhibition of microglial activation has emerged as a key therapeutic strategy. Rutin, a naturally occurring flavonoid glycoside abundantly, was found in plants such as buckwheat and citrus fruits. Rutin can inhibit neuroinflammation by decreasing gliosis and normalizing microglial NF-κB pathway. Rutin can directly decrease the production of pro-inflammatory cytokines of microglia caused by tau oligomers (Sun et al., 2021). NAD administration decreases microglial activation and neuronal death as well as alleviates production of ROS and pro-inflammatory cytokines (Zhao et al., 2021).

Modulating microglial phenotypic plasticity toward anti-inflammatory states represents another viable therapeutic avenue. Increasing M2 microglia and reducing M1 microglia can relieve neuroinflammation. Resveratrol activates silent information regulator 1 (SIRT1) to up-regulate the expression of peroxisome proliferator-activated receptor coactivator-1α (PGC-1α). Overexpression of PGC-1α suppresses the number of M1 phenotype but promotes M2 phenotype. And resveratrol and PGC-1α overexpression actively drive the M1-to-M2 phenotypic transition in microglia (Yang et al., 2017).

## 6 Conclusions and future prospects

Neuroinflammation is a vital participant in the initiation and progression of AD. The brain cannot be deemed as an immune-privileged organ. In the onset and development of AD, variations are detected in the receptors, physiological functions, and morphologies of microglia and astrocytes. Microglia and pro-inflammatory factors are the main therapeutic targets for the treatment of AD. One of the important goals of future studies is to explore the explicit molecular mechanisms regulating both microglia and astrocytes in neuroinflammation of AD. Further identification of agents involved in microglial activation is essential for precision-targeting of microglia therapy. In addition to drug therapy, promoting healthy habits could also be a potentially efficient therapeutic strategy for AD.
